# To defer or not to defer? A German longitudinal multicentric assessment of clinical practice in urology during the COVID-19 pandemic

**DOI:** 10.1371/journal.pone.0239027

**Published:** 2020-09-15

**Authors:** Nina N. Harke, Jan P. Radtke, Boris A. Hadaschik, Christian Bach, Frank P. Berger, Andreas Blana, Hendrik Borgmann, Florian A. Distler, Sebastian Edeling, Tobias Egner, Christina L. Engels, Mahmoud Farzat, Alexander Haese, Rainer Hein, Markus A. Kuczyk, Andreas Manseck, Rudolf Moritz, Michael Musch, Inga Peters, Sasa Pokupic, Bernardo Rocco, Andreas Schneider, André Schumann, Christian Schwentner, Chiara M. Sighinolfi, Stephan Buse, Jens-Uwe Stolzenburg, Michael C. Truß, Michael Waldner, Christian Wülfing, Volker Zimmermanns, Jörn H. Witt, Christian Wagner

**Affiliations:** 1 Department of Urology and Urologic Oncology, Hanover Medical School, Hanover, Germany; 2 Department of Urology, University of Duisburg-Essen, Essen, Germany; 3 Department of Urology, RWTH Aachen University, Aachen, Germany; 4 Department of Urology, University of Jena, Jena, Germany; 5 Department of Urology, Fürth Hospital, Fürth, Germany; 6 Department of Urology, Johannes Gutenberg University Medical Center, Mainz, Germany; 7 Department of Urology, Paracelsus Medical University, Nuremberg, Germany; 8 Department of Urology, Da Vinci Zentrum, Hanover, Germany; 9 Department of Urology, Klinikum Würzburg Mitte, Würzburg, Germany; 10 Department of Urology, Municipal Hospital Karlsruhe, Karlsruhe, Germany; 11 Department of Urology, Diakonie Klinikum, Siegen, Germany; 12 Martini-Klinik Prostate Cancer Center, University Clinic Hamburg-Eppendorf, Hamburg, Germany; 13 Department of Urology, Klinikum Magdeburg, Magdeburg, Germany; 14 Department of Urology, Klinikum Ingolstadt, Ingolstadt, Germany; 15 Department of Urology, Marien Hospital, Ruhr-University Bochum, Herne, Germany; 16 Department of Urology, Pediatric Urology and Urologic Oncology, Kliniken Essen-Mitte, Essen, Germany; 17 Department of Urology, Asklepios Klinikum Harburg, Hamburg, Germany; 18 Department of Urology, University of Modena and Reggio Emilia, Modena, Italy; 19 Department of Urology, Main-Kinzig-Kliniken Standort Gelnhausen, Gelnhausen, Germany; 20 Department of Urology and Kidney Transplantation, Martin Luther University, Halle (Saale), Germany; 21 Department of Urology, Diakonieklinikum Stuttgart, Stuttgart, Germany; 22 Department of Urology and Urologic Oncology, Alfried Krupp Krankenhaus, Essen, Germany; 23 Department of Urology, University of Leipzig, Leipzig, Germany; 24 Department of Urology, Klinikum Dortmund, Dortmund, Germany; 25 Department of Urology, St. Elisabeth Krankenhaus Köln-Hohenlind, Köln, Germany; 26 Department of Urology, Asklepios Klinik Altona, Hamburg, Germany; 27 Department of Urology, Siloah St. Trudpert Klinikum, Pforzheim, Germany; 28 Department of Urology, Pediatric Urology and Urologic Oncology, Prostate Center Northwest, St. Antonius Hospital Gronau, Gronau, Germany; Carolina Urologic Research Center, UNITED STATES

## Abstract

**Introduction:**

After the outbreak of COVID-19 unprecedented changes in the healthcare systems worldwide were necessary resulting in a reduction of urological capacities with postponements of consultations and surgeries.

**Material and methods:**

An email was sent to 66 urological hospitals with focus on robotic surgery (RS) including a link to a questionnaire (e.g. bed/staff capacity, surgical caseload, protection measures during RS) that covered three time points: a representative baseline week prior to COVID-19, the week of March 16^th^-22^nd^ and April 20^th^-26^th^ 2020. The results were evaluated using descriptive analyses.

**Results:**

27 out of 66 questionnaires were analyzed (response rate: 41%). We found a decrease of 11% in hospital beds and 25% in OR capacity with equal reductions for endourological, open and robotic procedures. Primary surgical treatment of urolithiasis and benign prostate syndrome (BPS) but also of testicular and penile cancer dropped by at least 50% while the decrease of surgeries for prostate, renal and urothelial cancer (TUR-B and cystectomies) ranged from 15 to 37%. The use of personal protection equipment (PPE), screening of staff and patients and protection during RS was unevenly distributed in the different centers–however, the number of COVID-19 patients and urologists did not reach double digits.

**Conclusion:**

The German urological landscape has changed since the outbreak of COVID-19 with a significant shift of high priority surgeries but also continuation of elective surgical treatments. While screening and staff protection is employed heterogeneously, the number of infected German urologists stays low.

## Introduction

On March 11^th^ 2020 the WHO declared the coronavirus disease 2019 (COVID-19) caused by SARS-CoV-2 (severe acute respiratory syndrome coronavirus 2) a pandemic. At that time, 1,567 cases were confirmed in Germany with three deaths due to COVID-19 after the first German patient was identified on January 27^th^ 2020 [1]. Germany is a federal state, and handling of the lockdown stayed in the responsibility of the individual states. Therefore, different steps were taken at different time points: the first states declared the lockdown in the week of March 16^th^ 2020. Measures included social distancing to “flatten the curve” based on simulation scenarios as well as drastic changes in the health care system [2]. In an efficient manner, triage systems and recommendations were developed by several international societies/associations to meet these challenges also in urology [3–5].

Based on those recommendations, both the healthcare system and the individual hospital had to respect different groups: infected patients, diagnosed and future urological patients as well as healthcare workers.

This study aims to give an insight to the urological situation and changes in surgical capacities, therapeutic and deferral strategies and management of staff and patients since the COVID-19 outbreak in Germany.

## Material and methods

### Online survey

On May 8^th^ 2020, 66 urological departments in Germany with a focus on laparoscopic/robotic surgery were contacted following an initiative of the German Association of Urology working group “Laparoscopy and robot-assisted surgery”. An email was send to the heads of the urological departments which included a link to an online questionnaire using the Google Docs open-source survey tool [6]. The survey was conducted in accordance with the Checklist of Reporting Results of internet-E-Surveys (CHERRIES) [7]. For successful completion of the survey, answering of all questions was not mandatory. A meticulous completion of the questionnaire was estimated to take 90 to 120 minutes. All members of our working group piloted the survey and no technical problems occurred. A similar survey was conducted by an Italian COVID-19 group and published by Rocco et al. [8].

The complete survey (S1 File) included detailed queries on numbers of available hospital beds and operating room (OR) capacity, staff members, surgical caseloads with subcategorization of surgeries at three different time points: week 1) baseline week that portrays the numbers of a regular/representative week before the outbreak of COVID-19, week 2) March 16^th^ to 22^nd^ 2020 which represent the first week after the lockdown in Germany (confirmed cases in Germany on March 16^th^ 2020: 6,012 with 13 deaths) and week 3) April 20^th^ to 26^th^ (confirmed cases in Germany on April 20^th^ 2020: 141,672; 4,404 deaths and approximately 91,500 recovered) [1]. For weeks 2 and 3, participants were asked about the rates of infected urologists and patients as well as protective measures during daily routine and robotic surgery (RS).

Preliminary results were presented during a webinar focusing on the development of RS after the outbreak of COVID-19 organized by the DGRU on May 14^th^ 2020, and participants were reminded to complete the questionnaire before the data collection was closed on May 24^th^ 2020.

### Daily situation report—Robert Koch Institute

The daily situation report of the pandemic in Germany and constantly updated case numbers of infected/recovered patients and death rates, including analysis of the 16 German federal states can be found on the homepage of the German Government´s agency for disease control and prevention, “Robert Koch Institute” (RKI) [1].

### Statistical analyses

SPSS 25 was used for statistical analysis. Categorial data are shown as frequencies and proportions; continuous variables are given as median and range. For improved visualization in the graphs, continuous data (e.g. number of staff members, cases) were summed to a total number with subsequent division to percentages. Chi-square and fisher’s exact test were used to identify differences in the the varying practice settings with a statistical significance p<0.05.

## Results

27 completed questionnaires were analyzed (41%) and included 14 responses from teaching hospitals, eight university hospitals and five non-academic centers. Each center (19 public and eight private) documented shifts in their numbers and capacities after the beginning of the pandemic compared to the representative baseline week before the outbreak.

### Developments in hospital capacities

**Fig 1:**
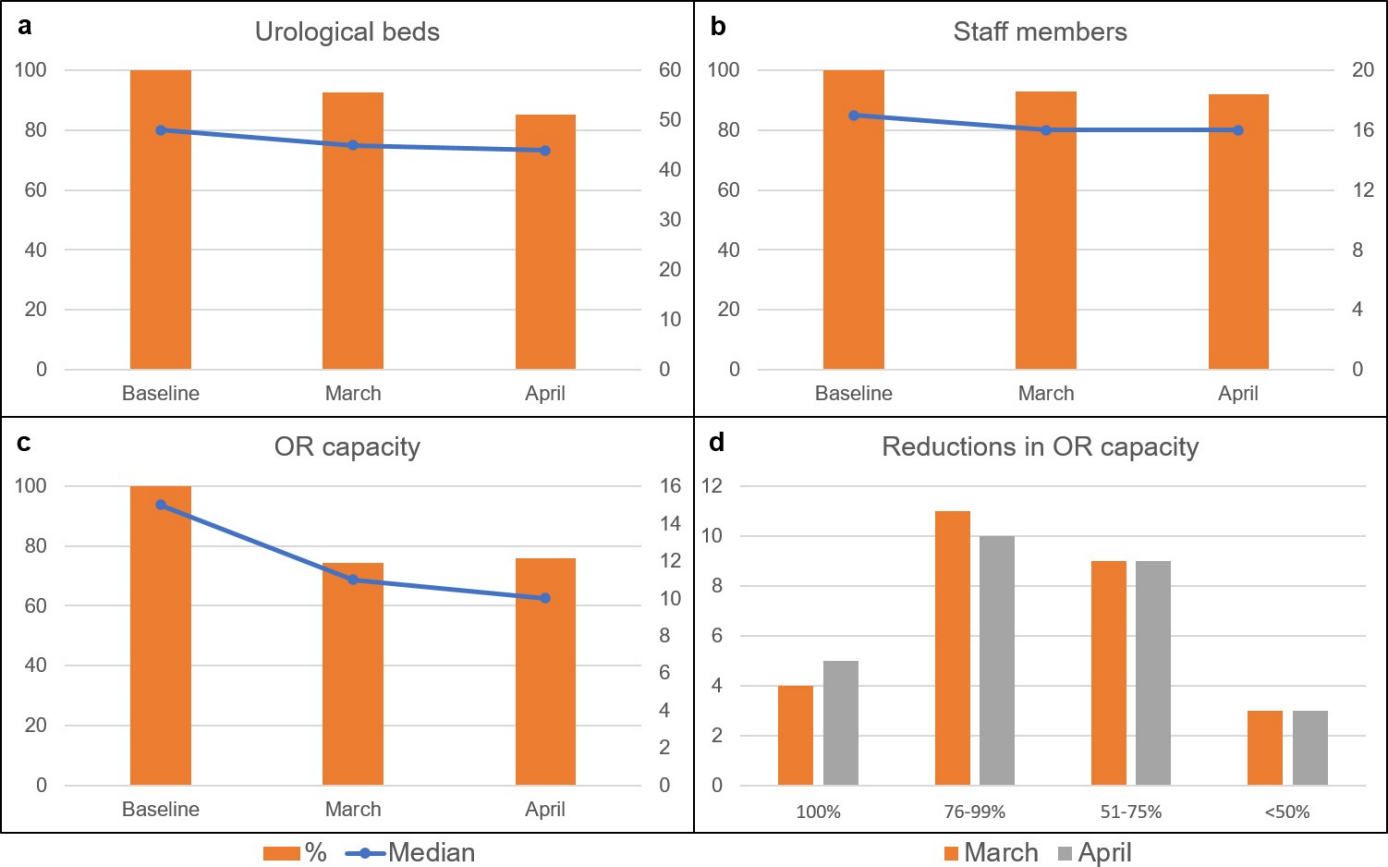
Changes in capacities. Trends in 27 urological departments (percentage change of total numbers (left y-axis, orange bars) and median number/center (right y-axis, blue line) for available beds (a), staff members (b) summed up operating rooms/week (c). D shows the portion of reductions in OR capacities in the different centers (y-axis).

While only a moderate cut-down of available beds within the different urological departments was found (median beds at baseline: 49 vs. 45 in March and 43 in April), the OR capacity was reduced distinctly by more than 25%, however this was handled heterogeneously and varied from a decrease to 22% of the initial capacity to no reduction. In total, 4571 surgeries were documented in the three requested weeks with 1195 emergency cases and 3376 scheduled cases. The number of scheduled patients per center declined from a median of 58 scheduled surgeries in the baseline week compared to 31 and 29 cases in March and April. In the same period, an increase of emergency cases could be observed with 363 in a regular week vs. 448 after the COVID-19 outbreak in March and 384 emergency surgeries in April (Fig 1).

No significant differences in management of OR capacities could be observed between university, teaching and non-academic hospitals in March (p = 0.53) and April (p = 0.29).

**Fig 2:**
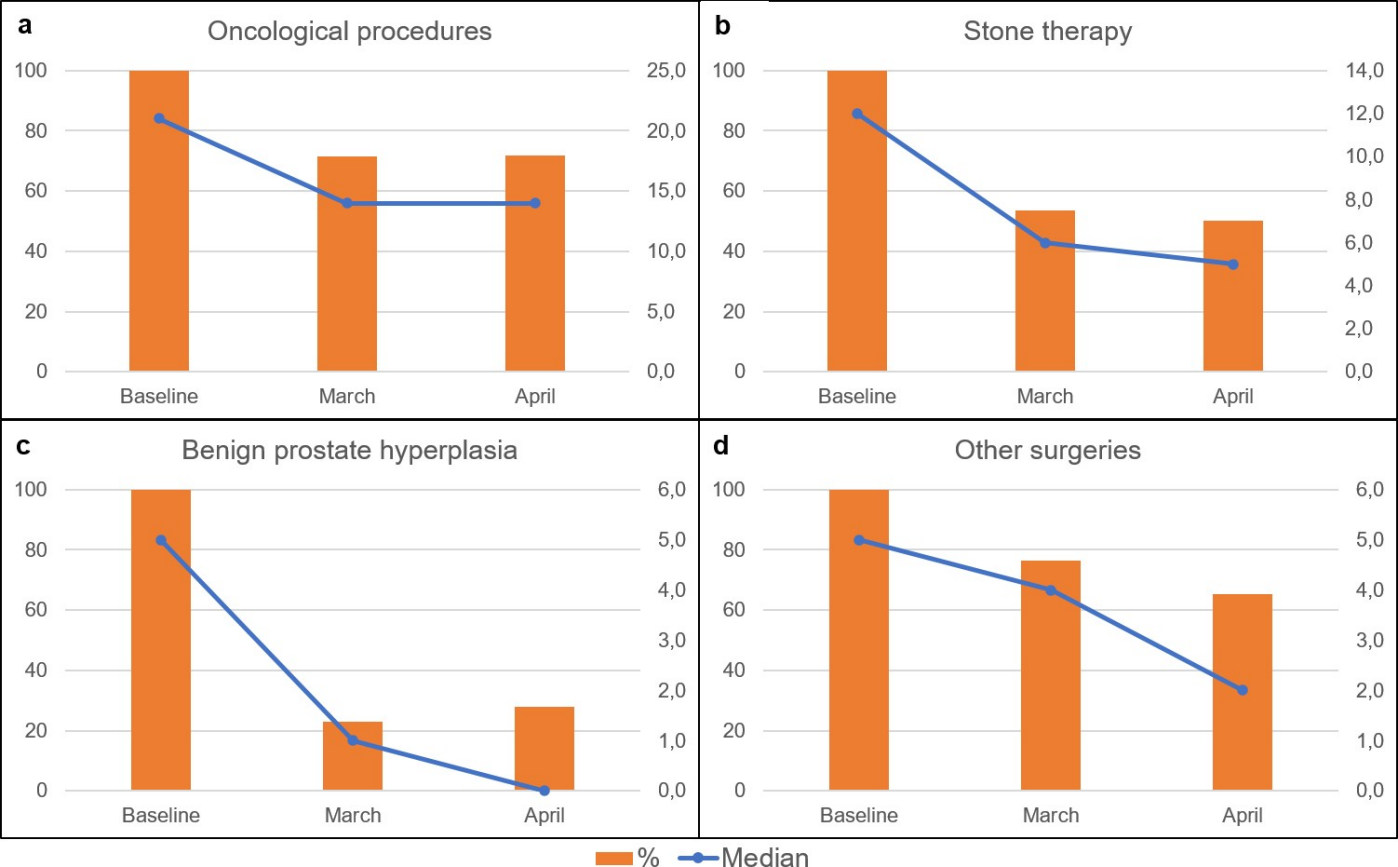
Changes in scheduled cases. Percentage change of total numbers (left y-axis, orange bars) and median number/center (right y-axis, blue line) for scheduled cases subdivided into oncological procedures (a), stone therapy (b), surgical treatment of benign prostate syndrome (c) and other surgeries (d).

The majority of scheduled cases in the baseline week represented oncological surgeries (44% of total scheduled cases) followed by urolithiasis (24%), surgical therapy of BPS (15%) and other surgeries (e.g. reconstructive, transgender, pediatric) with 18%. In the following weeks, declines could be observed in each of these subgroups (Fig 2).

**Fig 3:**
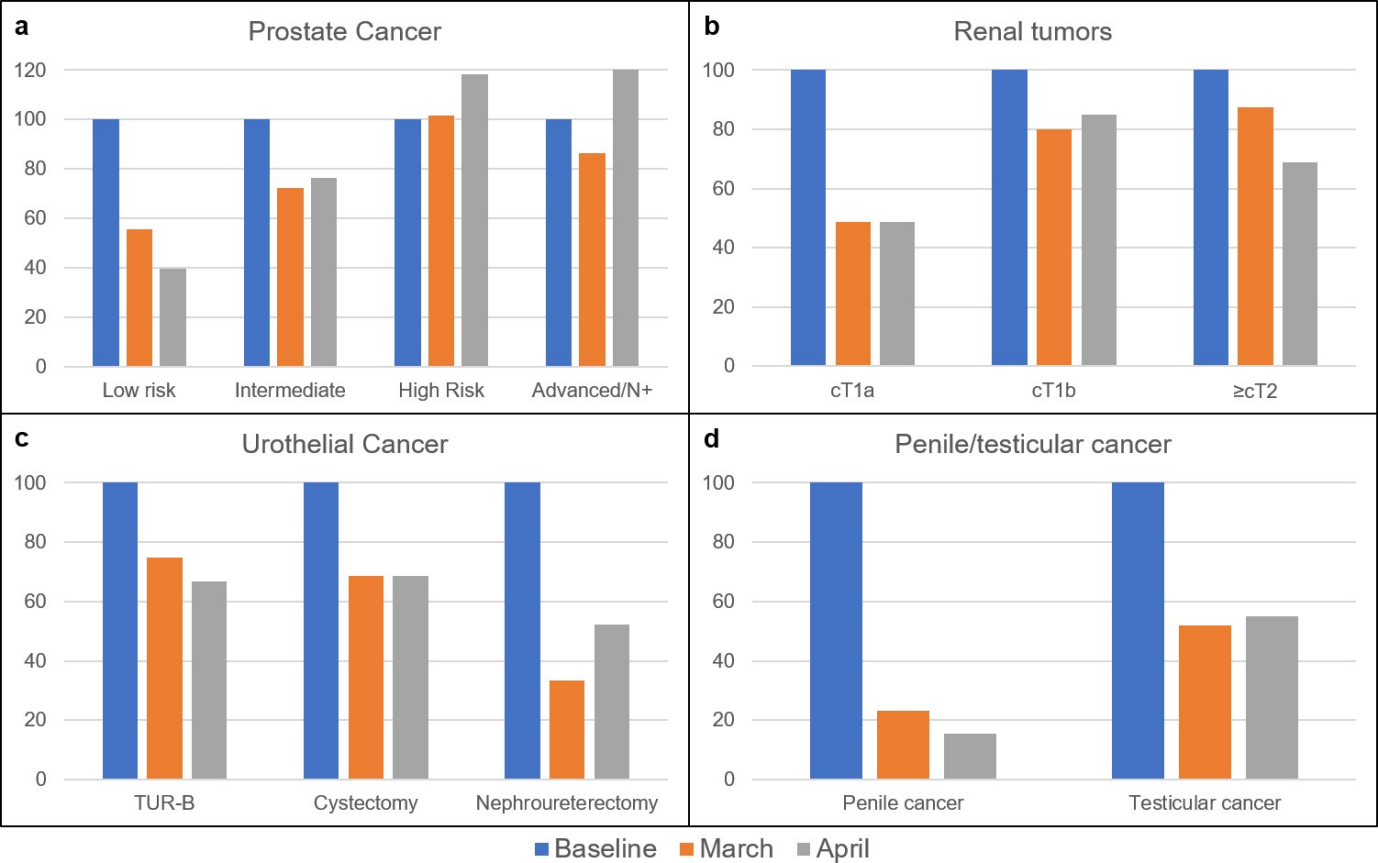
Changes in oncological surgeries. Percentage change of surgical therapy of prostate cancer after stratification according to the D’Amico score (a), renal masses with clinical stages (b), subgroups of procedures for urothelial cancer (c) and penile and testicular cancer (d).

Compared to the average numbers in the preceding weeks with a total of 366 cases, stone therapy (47% kidney and 53% ureteral stones) declined by 50%. Oncological surgeries were subclassified according to their priority and less urgent cancer therapies for low risk prostate cancer (baseline week: a total of 43 cases compared to 24 in March and 17 in April) and small renal masses (SRM) (reduction to 49% in March and April) were postponed, resulting in an increased or stable portion of surgeries for more advanced tumor constellations; the total number of prostatectomies for advanced prostate cancer rose from a total of 22 in the baseline week to 28 cases in April. Urothelial cancer treatment dropped from 297 cases to 211 in March and 195 in April with a comparable decrease for TUR-B and radical cystectomies. Remarkably, the rates of rare tumor entities including upper tract urothelial cancer as well as penile and testicular cancer decreased by 48–85% in comparison to the pre-COVID-19 case numbers (Fig 3).

### Robotic surgery in times of COVID-19

**Fig 4:**
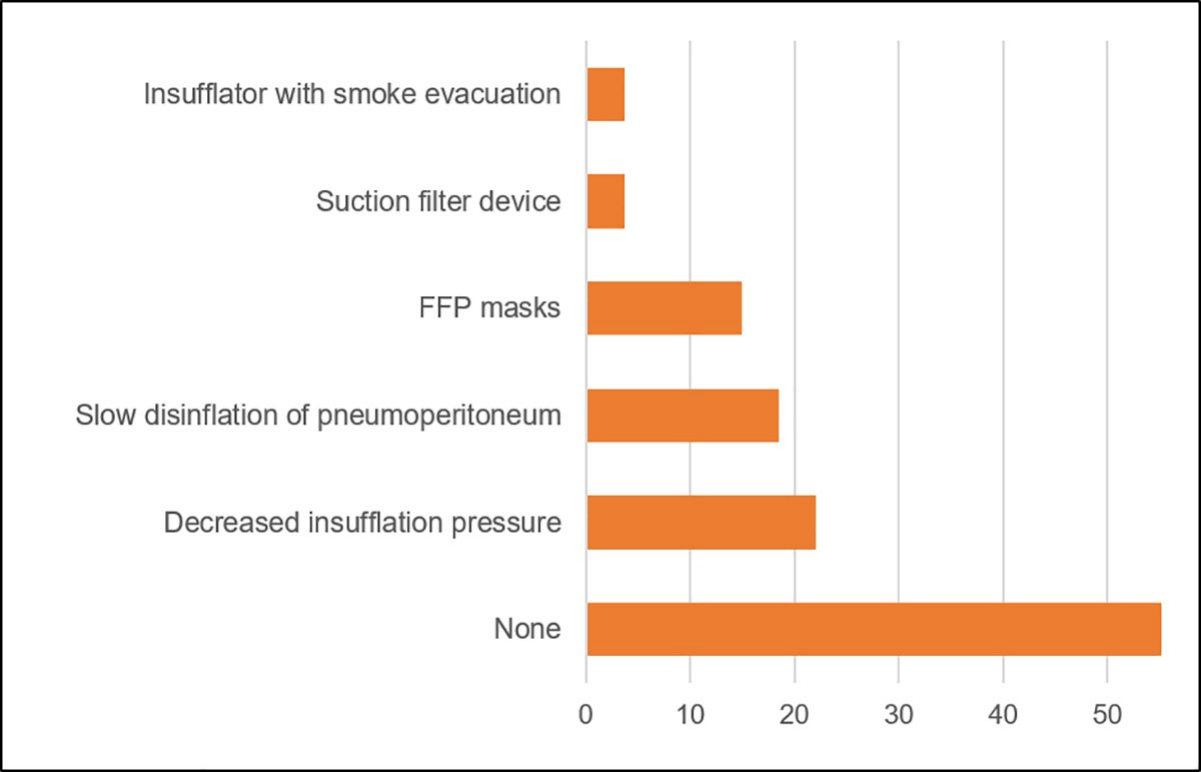
Protection during robotic surgery. Implementation of protection measures (in %) during robot-assisted surgeries in the different participating German centers.

In the pre-COVID-19 era, a median capacity of 4 robotic operating rooms/week was reported by the respondents ranging from two up to 20 robotic days/week with a maximum of 45 robotic cases in one high-volume center. Comparable to the developments for endourological (25%) and open/laparoscopic OR capacities (27%), robotic OR capacity decreased by 26% to a median of three days/week and a maximum of 12 robotic OR days/week in March but slowly increased to 83% of the initial capacity in April. Three centers suspended their urological robotic program in the weeks of March or April¸ one department paused RS completely since the outbreak. There was no difference in the general use of intraoperative safety precautions between public and private hospitals (42 vs. 50%, p = 1.00). Fig 4 summarizes the protective measures that were taken for RS.

### Patient’s screening

COVID-19 screening at the time of admission of patients comprised questionnaires in 78% of the institutions and physical examination for fever in 44%. In 52%, patients undergoing surgery were tested for COVID-19 prior to the operation with a comparable percentage in public and private hospitals (p = 0.21). A chest CT for screening was routinely employed in only one hospital. In seven departments patients were identified as COVID-19 positive either after screening (n = 3) or presentation of symptoms (n = 4).

### Staff management and safety precautions

To minimize the risk of virus transmission to the hospital staff, wearing of masks was required in all hospitals. This included regular surgical masks but also hand-sewn masks (19%). Regular screening of the staff with testing for COVID-19 was reported by 7% of the respondents and in only two urological departments the staff members’ temperature/fever was checked daily. To reduce the exposition to potentially infected patients, home office or (un)paid leave was enabled in 59% resulting in a reduction of the median staff members per center from 17 in the baseline week to 16 in March and April. Eight out of a total of 505 urologists (1.6%) were tested positive for SARS-CoV-2 infection.

## Discussion

When COVID-19 hit Europe, the impressive rates of infected patients and deaths in Italy as the first affected European country indicated that immediate and extraordinary steps were needed to face this threat. This resulted in unprecedented changes in the healthcare systems across the world. In hospitals, surgical capacity decreased significantly to reserve ventilators and ICU beds for COVID-19 patients. A global survey by Teoh et al. summarized a drop of 81–100% of outpatient clinic appointments and 48% of urological surgeries after the beginning of the pandemic [9]. To manage these reductions, new triage systems were implemented to identify those at high risk of COVID-19 and those at risk from being affected by delayed treatment of their urological disease. In April 2020 the European Association of Urology published recommendations in a remarkable short time frame based on the available information [3]. Surgeries should be scheduled only after risk stratification into one of four categories: low, intermediate, high priority and emergency. However, elective surgeries within the lowest risk category (including treatment of urolithiasis and BPS) were reported in March and in April. On the other hand, a 25% decrease of intermediate and high priority surgeries was also observed. This might be contributed to the differing policies of healthcare systems in the German federal states as well as hospital policies which may also explain the heterogeneous handling of OR capacities: while some respondents reported a reduction of more than 50%, others continued with a mostly unchanged program. Unsurprisingly, these findings for Germany are in line with a global survey that also revealed an inconsistent implementation of urological COVID-19 guidelines on procedure prioritization across the different practice settings and regions worldwide [10].

Major concern of the elective treatment of patients potentially harboring COVID-19 without clinical symptoms or initial negative testing have been raised after Lei et al. published results of 34 patients undergoing surgery while unknowingly being infected in January 2020 in Wuhan [11, 12]. All of these patients developed COVID-19 pneumonia with need for ICU care in 44% and a death rate of 21%. This disadvantageous postoperative course for COVID-19 positive patients was also supported by Nepogodiev [13]. The Diagnosis Related Group system in Germany determines a minimum hospital stay depending on the type of procedure. Major urological surgeries require a postoperative stay of at least 4–10 days, a time span that largely covers the incubation period of COVID-19 [14]. In a German study of 337 patients after radical prostatectomy (February to April 2020), no clinically evident COVID-19 infection occurred in the early postoperative time [15]. Accordingly, only a minority of the respondents reported COVID-19 positive patients in their departments in March and April.

The deferral of procedures and the fear of virus transmission in a urological department (outpatient or hospital) in combination with social distancing resulted in a further worrisome phenomenon: Novara et al. described a 55% decrease of urological consultations in emergency departments after the outbreak of the pandemic in Italy [16]. In contrast, our study confirmed an increase in urological emergency cases, like ureteral stent-placement, but we noticed a distinct reduction of TUR-Bs and primary surgical treatment of testicular cancer. Especially for the latter, this was surprising, as the reduced OR capacity was a rather negligible aspect; an orchiectomy is a short operation which can be done in a same-day surgery setting [17]. More likely, patients postponed an urological consultation for macrohematuria or a testicular nodule due to fear of a SARS-CoV-2 infection [18]. This is corroborated by further studies showing that the number of cardiac catherization for ST-elevation myocardial infarction decreased by 40 and 38% during the first weeks of COVID-19 [19, 20]. This observation in the context of a drop in prostate biopsy rates across Europe to 38% may indicate that a new wave of patients with possibly even more advanced disease might occur in the next months when outpatient care normalizes [21].

40% of the European participants of a global survey reported about voluntary or mandatory deployment to COVID-19 patient care and/or staff shortage [9]. Hence, in addition to continued high-quality patient care, protection of the healthcare workers is of utmost importance and the risk of virus transmission due to (unknown) contact with infected patients should be minimized. Our study shows a wide range of screening measures that was not always in line with the EAU recommendations: while some hospitals implemented triage admission wards with routine swabs on every patient, others relied solely on questionnaires.

Porter et al. suggested in a recent review that every patient should be managed as COVID-19 positive and one of the first recommendations in personal protection equipment (PPE) in the daily routine in and outside of the hospital is the use of a mask [22]. According to a German survey by Paffenholz et al. only a minority of respondents experienced a regular or continuous shortage of masks or gowns with no significant differences between university and regional hospitals [23]. In each of the 27 participating centers of the present study masks were included in the regular PPE. To adapt to a potential shortage of masks especially in the early days of the pandemic, some hospitals also used hand-sewn masks for patient care outside of the operating room.

Surgical teams are exposed to a high risk of contagion [24]. Especially in the initial phase with intubation and the preceding manual ventilation a high risk of contagion was described during the SARS (“bird flu”) outbreak in 2002 [25]. In the later period, surgical plume may endanger the operating staff as the generated aerosols may theoretically contain viable particles as shown for several viruses [26–28]. Hence, several surgical societies recommended a restrained use of laparoscopic or robotic procedures due to a possibly higher risk of aerosol generation [29, 30]. In our study we found an overall reduction in OR capacity by one fourth, but we did not observe a higher cut-back in robotic OR capacity compared to endourology/open procedures. Notably, less than 50% of the respondents reported taking special safety measures during robotic surgery, and only a minority routinely used FFP2/3 mask or a closed filtration system to protect the staff. This rather restricted intraoperative use of PPE can also be observed in other surgical specialties: routine PPE was reported by only 9.1% of the respondents of a global survey conducted by the International Society of University Colon and Rectal Surgeons [31] and solely 35% of minimally invasive emergency general surgeries in Italy were performed using measures for reduced aerosol dispersion [32]. However, with a rate of 1.6% SARS-CoV-2 positive (tested) urologists in our study this rather deviant inconsistent implementation of the “EAU Robotic Urology Section (ERUS) guidelines during COVID-19 emergency” [5] did not translate into a higher infection rate.

Several limitations have to be discussed. The response rate was below 50% possibly leading to a selection bias and the distribution of the online questionnaire focused on departments (sub)specialized on RS. Therefore, these findings cannot be generalized for institutions focusing on urolithiasis or reconstructive surgery as well as outpatient care. Furthermore, our results also reflect the differences in the German federal healthcare system and further analyses should elucidate the potential influence of varying states and hospital policies. Yet, in the present preliminary analysis, we observed no significant differences between varying practice settings (university, teaching and non-academic hospitals and private vs. public), a fact that was also confirmed globally [10].

The survey included detailed questions on capacities and case numbers but intentionally precluded further information on patient outcomes for ethical reasons. This initial study wanted to describe the situation in Germany during the first weeks of the pandemic and further studies are needed to elucidate the individual patient results before, during and hopefully after the COVID-19 era. However, in case of a continued pandemic, the evaluation of the changes in practice patterns and patient management during the first wave of COVID-19 and the subsequent impact on SARS-CoV-2 infection and death rates might be a foundation for future particularized and evidence-based guidelines to protect our patients and staff.

## Conclusion

The outbreak of COVID-19 has changed healthcare systems worldwide including the daily routine in urological departments. While several urological societies recommended postponement of elective therapies, we found a heterogeneous implementation in Germany with continuation of low priority cases as well as deferrals or shifts even of urgent surgeries. Screening strategies and staff protection is varying widely between the responding institutions, however, the numbers of infected German urologists and patients stayed low in our survey.

## Supporting information

S1 File Online questionnaire. The complete Google Doc survey included questions on capacities and surgical caseloads with subcategorization of surgeries at three different time points and protective measures and staff/patient management after the COVID-19 outbreak.
(DOCX)
