# Supplementary material for: To defer or not to defer? A German longitudinal multicentric assessment of clinical practice in urology during the COVID-19 pandemic
Source: PLoS One. 2020 Sep 15;15(9):e0239027. doi: 10.1371/journal.pone.0239027 (PMC7491711; doi:10.1371/journal.pone.0239027)
Supplement: S1 File — The complete Google Doc survey included questions on capacities and surgical caseloads with subcategorization of surgeries at three different time points and protective measures and staff/patient management after the COVID-19 outbreak. (DOCX) [file pone.0239027.s001.docx]

Covid-19 pandemic and urological surgery

Institution (name)

Country

City

Describe your Institution

1. Academic
2. Non-Academic

Describe your Institution

1. Public
2. Private

Referring week

1. Baseline Week
2. March 16^th^-22^nd^ 2020
3. April 20^th^-26^th^ 2020

Number of beds at the institution?

Number of beds in the urological department?

Number of urological staff members (urologists)?

How many OR days do you have in a regular week?

How many OR days do you have stratified by:

1. Endourology
2. Open/laparoscopic
3. Robot?

How many urgent interventions do you usually perform in a regular week?

1. Endourology
2. Open/trauma

How many patients do you have scheduled in a regular week stratified by

1. Oncology
2. Urolithiasis
3. BPS
4. Others?

How many patients with urothelial cancer?

1. TURB
2. Radical cystectomy
3. Nephroureterectomy

How many patients with prostate cancer?

1. D'Amico low risk
2. D'Amico intermediate risk
3. D'Amico high risk
4. Locally-advanced/nodal positive

How many patients with a renal tumor?

1. cT1a
2. cT1b
3. ≥cT2

How many patients with

1. Penile cancer
2. Testicular cancer?

How many patients with urolithiasis?

1. Renal stones
2. Ureteral stones

**Only for week 2 and 3**

How many urologists in your staff (urologists) were tested for COVID-19 in that week?

If someone was tested: due to symptoms or for screening?

How many urologists have been infected (laboratory-confirmed) in that week?

How many patients in your department have been infected in that week?

1. 0
2. 1-5
3. 6-10
4. 11-20
5. >20

Were patients tested due to symptoms or for screening?

**COVID Management**

Protective measures during robotic surgery

1. None
2. FFP2/3 masks
3. Insufflator with smoke evacuation
4. Decreased insufflation pressure
5. Slow disinflation of pneumoperitoneum
6. Suction filter device
7. Special operation rooms
8. Waiver of robotic surgery

Patient screening

1. None
2. Questionnaires (symptoms, exposition)
3. Temperature check
4. Nasopharyngeal swab testing for SARS-CoV-2
5. Serum tests for SARS-CoV-2
6. Chest CT

Staff management

1. Temperature check
2. Nasopharyngeal swab testing for SARS-CoV-2
3. Continuous wearing of masks
4. Use of hand-sewn masks
5. Arrangement of home office
6. Arrangement (un)paid leave
7. Arrangement of reduction of overtime hours
